# Editorial: Strategies to Improve Cardiac Function: Novel Ideas and Future Concepts

**DOI:** 10.3389/fcvm.2022.908331

**Published:** 2022-05-17

**Authors:** Palas K. Chanda, Junnan Tang, Ajit Magadum, Darukeshwara Joladarashi

**Affiliations:** ^1^Avance Biosciences, Houston, TX, United States; ^2^Department of Cardiology, The First Affiliated Hospital of Zhengzhou University, Zhengzhou, China; ^3^Key Laboratory of Cardiac Injury and Repair of Henan Province, Zhengzhou, China; ^4^Henan Province Clinical Research Center for Cardiovascular Diseases, Zhengzhou, China; ^5^Center for Translational Medicine, Lewis Katz School of Medicine, Temple University, Philadelphia, PA, United States

**Keywords:** stem cells, gene therapy, cardiac remodeling, heart failure, regeneration

Cardiovascular diseases (CVDs) remain a leading cause of annual death in the World. They emerge as a result of restriction or blockage in blood supply through blood vessels to heart muscles, a part of the brain, arms, and legs; or damage to heart muscles/valves and congenital disabilities that affect normal development and functioning of the heart. Limitations in the efficiency of conventional pharmacological and surgical approaches have led to translational studies based on molecular and cellular technologies, aiming to stimulate and or modulate endogenous molecular mechanisms responsible for CVDs. These studies are key in developing future strategies and methodologies to improve cardiac function. Although gene therapy is a major methodology used now-a-days for targeting therapeutic molecules at the protein level, mRNA technology is coming up rapidly as an alternative methodology after successfully developing the COVID-19 vaccine in 2020. Besides, as an alternative to these costlier methods, light-emitting diodes-based therapy (LEDT) is becoming popular because of its inexpensive, non-invasive, and easy-to-use method. This therapy has been widely used in recent years in multiple cardiac diseases. This low-energy therapeutic method does not cause any cardiomyocyte damage and has been shown to have anti-inflammatory effects in heart failure (HF). The present series of research articles on this Research Topic summarizes and discusses insightful concepts and different types of strategies to treat dysfunctions of the heart in CVDs. All these studies might be helpful to treat CVDs in the future. Here, we summarize the significant findings of interest to the readership and provide a frame of reference for future studies.

Changes in the cardiac microenvironment after myocardial ischemia (necrosis of cardiomyocytes, immune response and deposition of fibrotic scar tissue, etc.) lead to cardiac remodeling, which can gradually progress to heart failure. Reversing pathological ventricular remodeling can start from the cardiomyocytes themselves, including reducing cardiomyocyte apoptosis, reducing cardiomyocyte hypertrophy, or modulating other cellular states in cardiac tissue, such as promoting endothelial cell migration, promoting angiogenesis, and regulating inflammatory cells infiltration or polarization of macrophages reduces excessive inflammatory response, etc. Kumari et al. reported that ALKBH5 helps in the maintenance of angiogenesis in endothelial cells following acute ischemic stress via reduced SPHK1 m6A methylation and downstream eNOS-AKT signaling, which provide insight into the novel strategy for angiogenesis. In addition, the Wang group found that Lysosomal-associated protein transmembrane 5 (LAPTM5) could be involved in pathological cardiac hypertrophy through the MEK-ERK1/2 pathway, the antihypertrophic effect of LAPTM5 could be blocked by constitutively active mutant Rac1 (G12V), which shed light on therapeutic potential in the treatment of pathological cardiac hypertrophy. Notably, organelles (i.e., lysosomes, endoplasmic reticulum, etc.) and adipose tissue may be involved in the pathological process after ischemia injury. Cation-independent mannose 6-phosphate receptor (Cl-MPR), a critical protein tag for the proper transport of lysosomal contents, is decreased and the distribution of lysosomal cathepsins is abnormal when cardiomyocytes were impaired with ischemia/hypoxia (I/H). TBC1D5 could be an essential regulator of the distribution of lysosomal cathepsins during the I/H process (Yuesheng Huang et al.). Also, the Dong group illustrated that Seipin, an endoplasmic reticulum (ER) membrane protein, plays a critical role in maintaining perivascular adipose tissue function and vascular homeostasis (Jianzeng Dong et al.). Apart from the therapeutic strategies to block the pathological process, bioengineered treatments such as biomaterials and optical technologies also play an important role in repairing cardiac injury. A study conducted in a mouse model showed that LED-Red (630 nm) therapy significantly increases ATP level and calcium handling in the HF heart, both of which are important as an energy source and to maintain contractility. Besides, this therapy also attenuated perivascular fibrosis and collagen deposition. Thus, this therapy seems to improve the overall cardiac function under HF condition.

Diabetes mellitus plays a crucial role in accelerating the formation and rupture of atherosclerotic plaques, promoting myocardial fibrosis, and reducing cardiac function. Patients with diabetic cardiomyopathy (DCM) are highly prone to adverse cardiovascular events and significantly increased risk of death. The mechanisms underlying DCM pathogenesis and effective treatments need to be further explored. The STK35 is reduced in the diabetic human heart, and also mouse cardiac endothelial cells treated with high glucose. Intravenous injection of AAV9-STK35 viral particles in diabetic mice leads to increased vascular density, suppression of fibrosis in the heart, and amelioration of left ventricular function (Wang et al.). STK35 could be a potential gene therapy target for treating DCM.

Chronic thromboembolic pulmonary hypertension (CTEPH) is a progressive pulmonary vascular disease with a poor natural prognosis; the median 2–3-year survival rate of patients with diagnosed CTEPH is as low as 10–20% without timely intervention. Balloon pulmonary angioplasty could be effective for CTEPH, but the evaluation criteria for surgical prognosis are still lacking. Li et al. found that measuring diffusing capacity for carbon monoxide (DLCO) dynamically facilitates identifying patients who might have unsatisfactory hemodynamic results after BPA.

In summary, this Research Topic addressed new strategies and novel ideas to treat heart functions and targeted therapy of CVDs, which are expected to reduce the morbidity and mortality of patients with CVDs. We believe that this Research Topic allows readers to fully appreciate the importance of the various strategies and ideas used to improve cardiac functions.

## Author Contributions

All authors listed have made a substantial, direct, and intellectual contribution to the work and approved it for publication.

## Conflict of Interest

PC is the Director of Avance Biosciences. The remaining authors declare that the research was conducted in the absence of any commercial or financial relationships that could be construed as a potential conflict of interest.

## Publisher's Note

All claims expressed in this article are solely those of the authors and do not necessarily represent those of their affiliated organizations, or those of the publisher, the editors and the reviewers. Any product that may be evaluated in this article, or claim that may be made by its manufacturer, is not guaranteed or endorsed by the publisher.

